# Decolorization applicability of sol–gel matrix immobilized manganese peroxidase produced from an indigenous white rot fungal strain *Ganoderma lucidum*

**DOI:** 10.1186/1472-6750-13-56

**Published:** 2013-07-13

**Authors:** Hafiz Muhammad Nasir Iqbal, Muhammad Asgher

**Affiliations:** 1 Industrial Biotechnology Laboratory, Department of Chemistry and Biochemistry, University of Agriculture, Faisalabad, Pakistan

**Keywords:** Bio-catalysis, *G*. *lucidum*, MnP, PAGE, Sol–gel, Immobilization, Textile effluents, Decolorization, Toxicity reduction

## Abstract

**Background:**

An eco-friendly treatment of industrial effluents is a major environmental concern of the modern world in the face of stringent environmental legislations. By keeping in mind the extensive industrial applications of ligninolytic enzymes, this study was performed to purify, and immobilize the manganese peroxidase (MnP) produced from an indigenous strain of *Ganoderma lucidum*. The present study was also focused on investigating the capability of immobilized MnP for decolorization of dye containing textile effluents.

**Results:**

A large magnitude of an indigenous MnP (882±13.3 U/mL) was obtained from white rot fungal strain *G*. *lucidum* in solid state bio-processing of wheat straw under optimized fermentation conditions (moisture, 50%; substrate, 5 g; pH, 5.5; temperature, 30°C; carbon source, 2% fructose; nitrogen source, 0.02% yeast extract; C: N ratio, 25:1; fungal spore suspension, 5 mL and fermentation time period, 4 days). After ammonium sulfate fractionation and Sephadex-G-100 gel filtration chromatography, MnP was 4.7-fold purified with specific activity of 892.9 U/mg. *G*. *lucidum* MnP was monomeric protein as evident by single band corresponding to 48 kDa on native and denaturing SDS-PAGE. The purified MnP (2 mg/mL) was immobilized using a sol–gel matrix of tetramethoxysilane (TMOS) and proplytrimethoxysilane (PTMS). The oxidation of MnSO_4_ for up to 10 uninterrupted cycles demonstrated the stability and reusability of the immobilized MnP. Shelf life profile revealed that enzyme may be stored for up to 60 days at 25°C without losing much of its activity. To explore the industrial applicability of MnP produced by *G*. *lucidum*, the immobilized MnP was tested against different textile effluents. After 4 h reaction time, the industrial effluents were decolorized to different extents (with a maximum of 99.2%). The maximally decolorized effluent was analyzed for formaldehyde and nitroamines and results showed that the toxicity parameters were below the permissible limits.

**Conclusions:**

In conclusion, *G*. *lucidum* MnP was immobilized by sol–gel matrix entrapment with an objective to enhance its practical efficiencies. The MnP was successfully entrapped into a sol- gel matrix of TMOS and PTMS with an overall immobilization efficiency of 93.7%. The sol- gel entrapped MnP seems to have prospective capabilities which can be useful for industrial purposes, especially for bioremediation of industrial effluents.

## Background

To date phenolic/non-phenolic compounds and some toxic environmental pollutants particularly textile waste water effluents posing serious health hazards to the entire living ecosystem and especially on the animals and humans. Over the past several years, there has been great interest among researchers in the production of ligninolytic- and cellulose-degrading enzymes from various agro-industrial waste materials and their by-products, such as wheat straw, rice husk, banana waste, citrus peel, rice straw, corncobs, corn stover, apple pomace, and sugar cane bagasse [1-8]. These wastes are not properly disposed off in developing countries and have become a major source of ecological pollution. Significant efforts have been made to convert lignocellulosic residues to valuable products such as bio-fuels, chemicals and animal feed with the help of ligninolytic enzymes of WRF, many of which have been successful [2,4-8]. To degrade all the components of lignocellulosic materials and phenolic organic substrates, white rot fungi (WRF) are so far exclusive in their strong oxidative capabilities due to their extracellular nonspecific enzyme system [3].

WRF produce two major families of enzymes, generally termed ligninolytic enzymes, *i*.*e*., extracellular peroxidases (manganese peroxidase, MnP; manganese independent peroxidase, MIP; lignin peroxidase, LiP; and versatile peroxidase, VP) and phenol oxidases (laccases). MnP and laccases have been applied frequently in various bio-technological processes such as lignocellulosic biomass delignification for ethanol production, oxidation of pollutants, bioremediation processes, textile bio-finishing, beverage processing, bio-bleaching of pulps and detergent manufacturing [1-3,6-8]. MnPs are heme-containing glycoproteins having capability to catalyze the oxidation of Mn^2+^ to Mn^3+^. The Mn^3+^−organic acid complexes, in turn, oxidize phenolic structures present in lignin and also various lignin related organic compounds [9]. In nature, MnP catalyzes plant lignin de-polymerization as component of ligninolytic enzymes complex. A wide range of substrate oxidizing capability renders it an interesting enzyme for biotechnological applications in several industries.

Textile waste effluents contain several types of chemicals including real dyes itself that are toxic, carcinogenic or mutagenic. Textile industries discharged their waste effluents into water streams with or without some partial treatments which mainly causes water pollution and also dangerous for aquatic life. In literature, various physical/chemical methods have been reported by several authors to effectively process the textile effluents such as adsorption, precipitation, chemical reduction, ionizing radiations and ultra filtration [4,5,10]. In spite of the existing physical/chemical technologies for colour removal which are usually expensive and commercially/environmentally unattractive, biological processes provide an alternative to existing physico−chemical technologies because they are cost effective, eco−friendly and can be applied to wide range of dye containing industrial effluents [2,4,5].

Enzyme immobilization offers a noteworthy solution to industrial and environmental challenges faced by the modern world predominantly in the applications of enzymes in agro-waste management, textile effluents decolorization, and alternatives of health hazardous chemical based procedures. To date, various techniques have been reported for enhancing activity and operational stability of industrial enzymes. Gel entrapment is preferred over other immobilization techniques as this method is convenient and the structure of the enzyme remains secure. Recently, sol−gels have attracted the attention of biotechnologists due to their ability to produce enzymes in defined thin films that are thermo-stable and non-toxic in nature [2,4]. Among the various potent lignin-degrading microorganisms, *G*. *lucidum* is a potential MnP producer with high catalytic potentials that are suitable for a wide range of environmental/biotechnological applications. Therefore, in the current study MnP from *G*. *lucidum* was immobilized using sol–gel matrix entrapment technique. The decolorization of different textile effluents by the sol–gel matrix entrapped MnP was also the main focus of the present study to present its approach for bioremediation applications.

## Results and discussion

### Production, purification and immobilization of MnP

**Figure 1:**
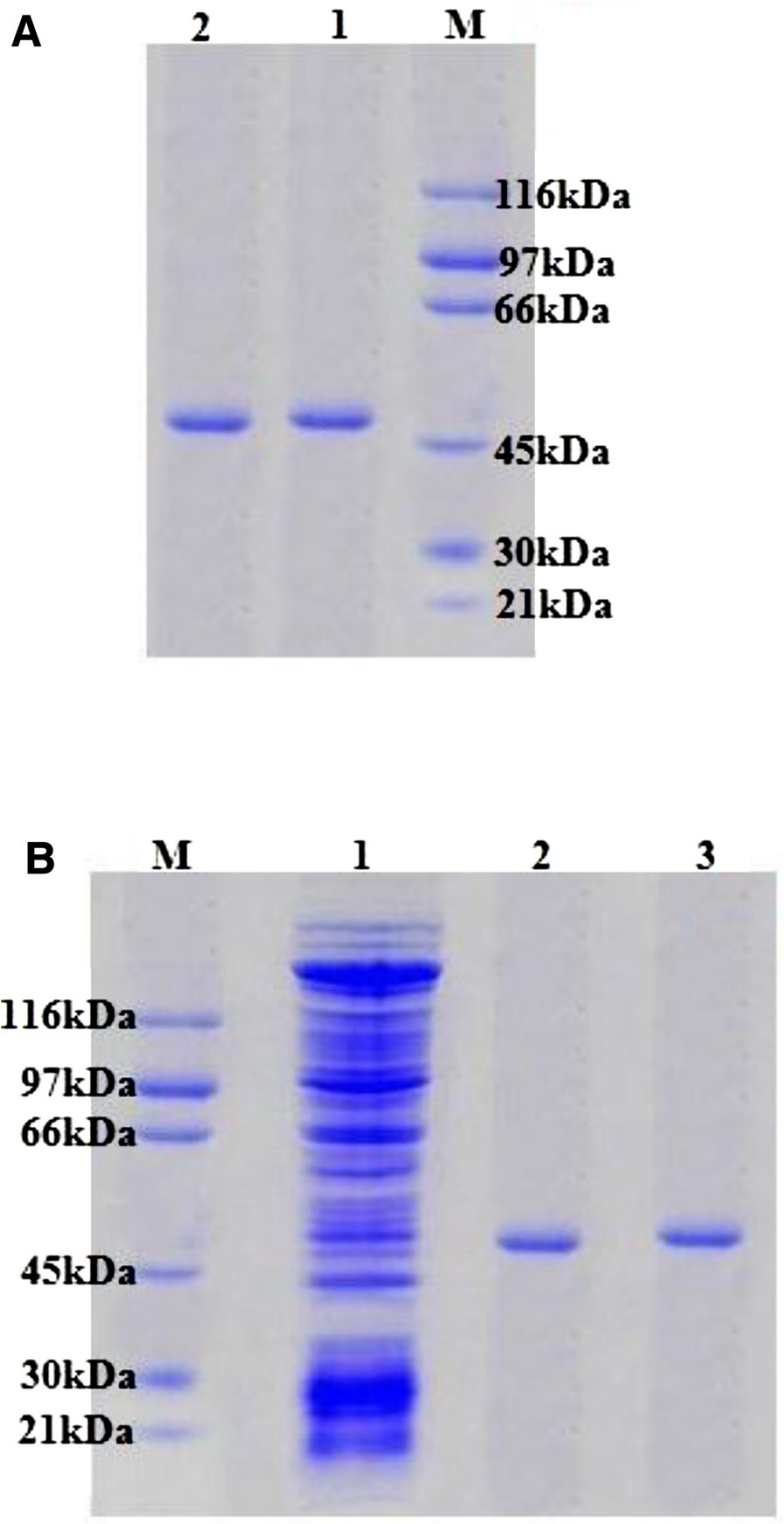
**Native (A) and SDS (B) PAGE for MnP produced by *G*. *lucidum*.**

A large magnitude of an indigenous MnP (882±13.3 U/mL) was obtained from white rot fungal strain *G*. *lucidum* in solid state bio-processing of wheat straw under optimized fermentation conditions (moisture, 50%; substrate, 5 g; pH, 5.5; Temperature, 30°C; Carbon source, 2% fructose; nitrogen source, 0.02% yeast extract; C: N ratio, 25:1; fungal spore suspension, 5mL and fermentation time period, 4 days). Active MnP fraction was 4.7-fold purified by Sephadex G-100 gel filtration chromatographic technique with an overall yield and specific activity of 8.1% and 892.9 U/mg, respectively (Table 1). Native and SDS-PAGE was used to confirm the purity of MnP at homogeneity level corresponding to its single peaked band of 48 kDa (Figure 1). The purified MnP was immobilized using sol–gel matrix entrapment technique with 93.7% immobilization efficiency using the fraction containing 2 mg/mL MnP. Previously, the purification protocols involving Sephadex-G-100 gel filtration technique for the purification of various fungal enzymes, including cellulases, protease, laccase, MnP and LiP from different microbial cultures have been developed [2-5,11]. In addition, the results obtained by the sol–gel entrapment for the present MnP immobilization are superior to those reported for covalent binding of the enzyme on siliceous cellular foams, sepa beads and amine-terminated magnetic nano−composite by glutaraldehyde cross linking method [12,13]. However, the reported covalent binding strategies involving a coupling reagent such as glutaraldehyde are much more expensive as compare to the sol–gel matrix entrapment method.

**Table 1:** **Purification summary for MnP produced by *G*. *lucidum***

**Purification steps**	**Total volume (mL)**	**Total enzyme activity (U)**	**Total protein content (mg)**	**Specific activity (U/mg)**	**% Yield**	**Purification fold**
Crude enzyme	200	176400	925	190.7	100	1
(NH_4_)_2_SO_4_	25	22875	107	213.8	12.9	1.1
Precipitation						
Dialysis	20	19500	64	304.7	11.1	1.6
Sephadex-G-100	13	14287	16	892.9	8.1	4.7

### Reusability and shelf life

**Figure 2:**
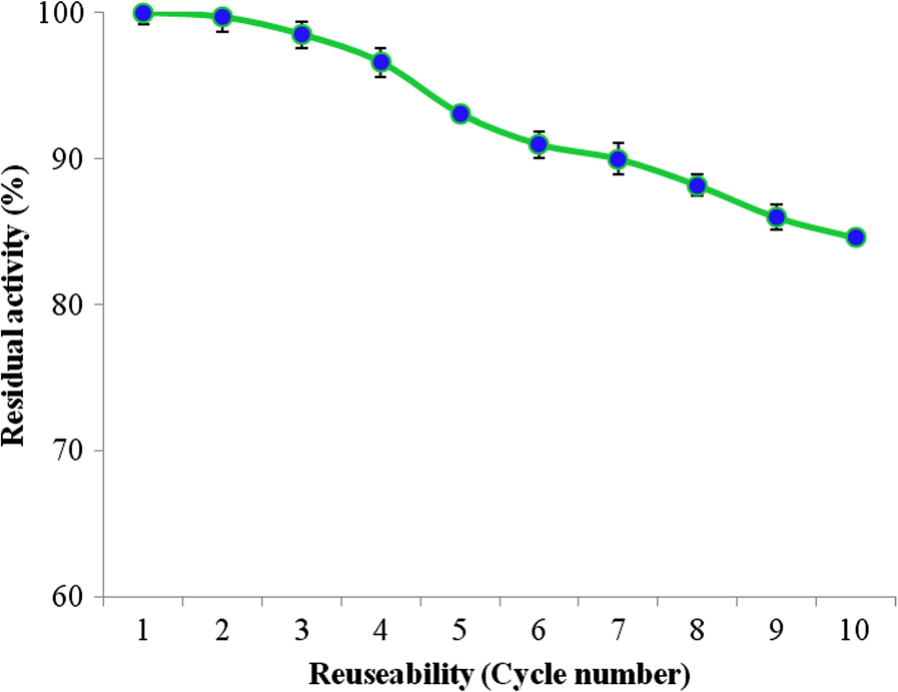
Reusability of sol–gel matrix entrapped MnP after ten repeated cycles.

**Figure 3:**
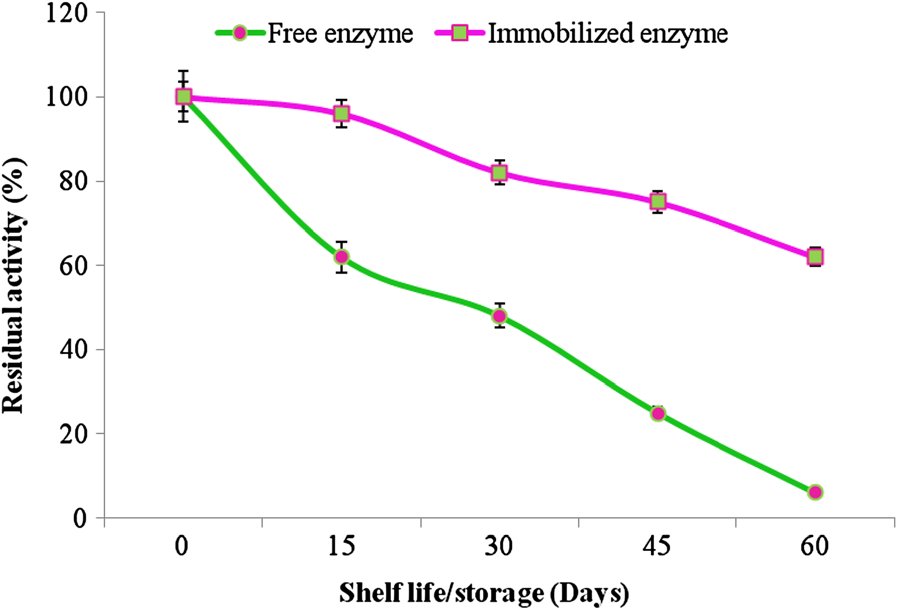
Shelf life/storage of free and sol–gel matrix entrapped MnP.

Figure 2 illustrated that the sol–gel entrapped MnP retained up to 84.6% of initial activity after 10 periodic oxidation cycles of MnSO_4_. The effect of storage on the activity of free and entrapped MnP was determined by incubating at room temperature (25°C) for up to 75 days. Sol–gel entrapped MnP was more stable (68% higher) than that of the free MnP at room temperature (Figure 3). In comparison to the present study, previously Yinghui et al. [14] reported a residual activity of 60% only after 10 repeated batches for the covalently immobilized laccase while in another study, Xiao et al. [12] reported a residual activity of 80% after five consecutive batches. Kunamneni et al. [13] observed that the storage stability of *M*. *thermophila* laccase immobilized on Sepabeads EC-EP3 carriers was 5% higher as compare to that of the free laccase.

### Decolorization of textile effluents by immobilized MnP

**Figure 4:**
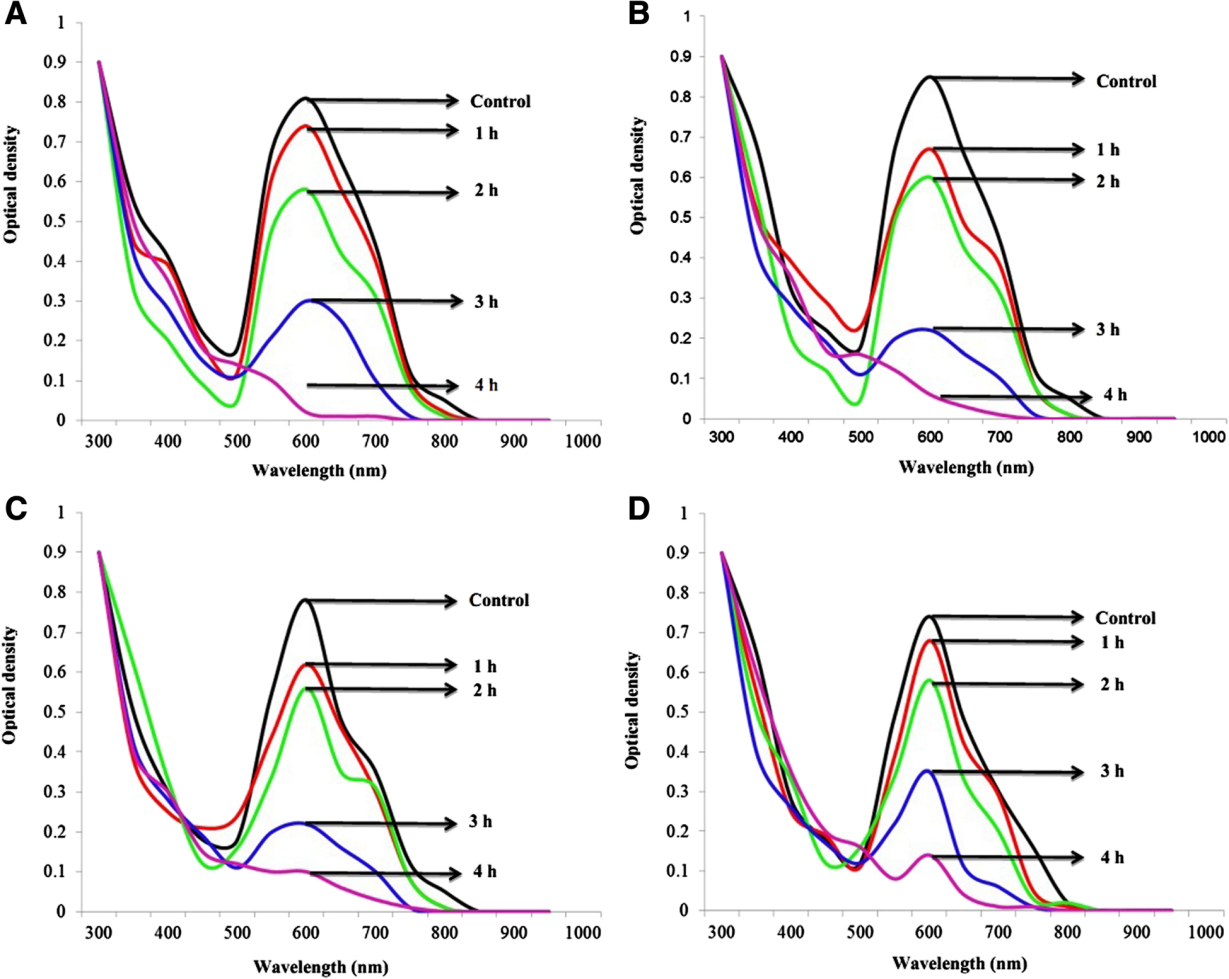
**UV–vis absorption spectra of Crescent textile effluent (A), Magna textile effluent (B), Arzoo textile effluent (C) and Chenab textile effluent (D) obtained after treatment with *G*. *lucidum *MnP.**

After 4 h reaction time under continuous shaking batch, the sol–gel- immobilized MnP was found to maximally decolorize the Crescent textile effluent (99.2%) (Figure 4A) followed by Magna textile effluent (94.6%) (Figure 4B), Arzoo textile effluent (89.6%) (Figure 4C), and Chenab textile effluent (78.5%) (Figure 4D). An increase in the effluent decolorization was observed with an increase in reaction time and maximum was found after 4 h reaction time in a temperature controlled batch culture shaking environment. WRF grown in synthetic textile dye solutions and industrial effluents take more time to decolorize dyes, compared to isolated enzymes, because of the necessary lag phase before they grow and secrete ligninolytic enzymes for dye degradation. Recently, in another effluent decolorization study, we have noted a significant time reduction and effluent colour loss (98.47%) in 48 h by the addition of MnSO_4_, followed by ABTS and varatryl alcohol as mediators for MnP, laccase and LiP respectively [8]. The variation in effluent composition is also responsible for variation in its decolorization by enzyme extracts from different fungi [15].

### Toxicity studies

#### Nitro-amines and formaldehyde

Traces of nitro-amines were found in the maximally decolorized treated sample, indicating that nitro-amines were produced as intermediates during effluent decolorization. The formaldehyde content was 12.2 mg/L for untreated effluent and it reduced to 3.6 mg/L after treating with the enzyme. Significantly lower quantities of nitro-amines and formaldehyde in the treated effluents suggest their formation as intermediates in the mechanism of degradation of dyes and other chemical compounds present in the effluent that were ultimately completely degraded by immobilized MnP. The ligninolytic enzymes enhance the aerobic degradation/ mineralization of dyes and pollutants that does not result in the formation of aromatic ammines [16]. The results of the toxicity studies showed that the effluents were not only decolorized but also detoxified by the action of immobilized MnP.

## Conclusions

*G. lucidum* MnP was immobilized by entrapping in a sol–gel matrix with an objective to enhance its functionalities. The MnP was successfully entrapped into a sol–gel matrix of TMOS and PTMS with maximum of 93.7% immobilization efficiency. The sol–gel entrapped MnP presented potential efficiency as bio-catalyst for decolorization of dye containing textile effluents and it was eco-friendly, chemical free and energy saving approach for bio-remediation on textile industry effluents.

## Methods

### Fungal culture, chemicals, and agro-waste substrate

A pure white rot fungal strain *G*. *lucidum* obtained from fungal culture collection of Industrial Biotechnology Laboratory, Department of Chemistry & Biochemistry, University of Agriculture Faisalabad, Pakistan, was used for production of MnP in SSF. Sephadex-G- 100, Polyvinyl alcohol, TMOS, and PTMS were from Sigma-Aldrich (USA), while all other chemicals were of analytical grade and used as such. The lignocellulosic waste wheat straw was collected from the Student Research Farms, University of Agriculture Faisalabad, Pakistan. To avoid moisture, the substrate was oven dried (60°C), ground to fine particle size (40 mm mesh size) using Wiley Mill (electric grinder), and stored in airtight plastic jars. For decolorization studies, four textile effluents were collected from Magna, Crescent, Arzoo, and Chenab textile industries of Faisalabad, Pakistan.

### Fungal spore suspension

To develop homogeneous fungal spore suspension (10^7^-10^8^ spores/mL), *G*. *lucidum* was cultivated at 30±1°C for 5 days in an Erlenmeyer flask containing a basal salt medium. The constituents of the medium were: (NH_4_)_2_SO_4_, 10 g/L; KH_2_PO_4_, 4 g/L; MgSO_4_·7H_2_O, 0.5 g/L and CaCl_2_, 0.5 g/L. Before fungal inoculation, the medium was sterilized at 121°C and 1.035 bar pressure in a laboratory scale autoclave (Sanyo, MLS-3020U, Japan) for 15 min.

### MnP production and extraction protocol

MnP production by *G*. *lucidum* was carried out in 500 mL capacity Erlenmeyer flasks using wheat straw under optimized growth conditions. The optimized conditions were: moisture, 50%; substrate, 5g; pH, 5.5; temperature, 30°C; carbon source, 2% fructose; nitrogen source, 0.02% yeast extract; C: N ratio, 25:1; fungal spore suspension, 5mL; fermentation time, 4 days. Triplicate flasks were autoclaved (120°C) and inoculated with 5 mL freshly prepared fungal inoculum. The inoculated flasks were kept at 30°C in a still culture incubator (EYLA SLI-600ND, Japan) for 4 days. MnP was extracted by adding 100 mL of distilled water to the 4 day old fermented solid-state cultures, followed by shaking at 120 rpm for 30 min. The contents were filtered through Whatman No.1 filter paper and the filtrates were centrifuged at 3,000 g for 10 min. The supernatants were carefully collected and used as crude MnP extracts for MnP assay, purification, and sol–gel immobilization.

### MnP activity and protein contents determination

UV/Visible spectrophotometric method was used to determine the activity of MnP as described earlier [3,17]. Assay reaction mixture contained 1 mL of 1 mM MnSO_4_ as a substrate, 1 mL of 0.5 mM sodium malonate buffer of pH 4.5, 0.5 mL of 0.1M H_2_O_2_ and 0.1 mL of enzyme solution. Blanks contained the same mixture solution without enzyme. Absorbance of each sample was taken after 10 min interval at 270 nm using UV-Vis spectrophotometer (T60, PG Instruments UK). Unit activity was defined as the amount of enzyme required to produce a unit increase in absorbance at specific wavelength (nm) per mL of reaction mixture. Bradford micro assay was used with bovine serum albumin as standard to determine the protein contents of each sample [18].

### Purification and PAGE

Crude MnP extract obtained from solid state culture of *G*. *lucidum* was centrifuged (3,000 g) for 15 min at 4°C to attain maximum clarity, and the supernatant was concentrated by freeze drying. The crude enzyme concentrate was placed in ice bath and crystals of ammonium sulfate were added to attain 60% saturation followed by the centrifugation at 5,000 g for 30 min at 4°C. The pellets were dissolved in 50 mM malonate buffer and dialyzed against the same buffer at room temperature with 4 equal changes of buffer after every 6 h to remove extra salt. Total proteins and MnP activity were determined before and after dialysis as described previously. The dialyzed active fractions were loaded on Sephadex G-100 column (2×25 cm) for further purification. Up to 20 active fractions each of 1mL were collected with flow rate of 0.5 mL/min and monitored for MnP activity as described before. Native and SDS poly acrylamide gel electrophoresis was performed according to the method of Laemmli [19], using Mini-gel electrophoresis apparatus (V-GES, Wealtec Corp. USA). The molecular mass of the purified MnP was estimated in comparison to standard molecular weight markers (standard protein markers, 21-116kDa; Sigma, USA). The protein bands were visualized by staining with Coomassie Brilliant Blue G-250 (Sigma, USA).

### Sol–gel immobilization

To prepare the sol-gel thin films for enzyme entrapment purposes, TMOS and PTMS were used in molar TMOS: PTMS (T: P) ratios of 1:2 ratio by adopting the methodology as described earlier by Asgher and Iqbal [2]. A purified active MnP fraction (2 mg/mL) was suspended in de-ionized water and centrifuged (4,000 g) for 15 min at 4°C. The separated supernatant fluid was added to an equal ratio mixture of aqueous sodium fluoride, polyvinyl alcohol, and de-ionized water. The solution was shaken and PTMS was added, followed by the addition of TMOS. The reaction mixture was gently mixed for 20 sec in a vortex mixer and placed in an ice bath until gelation occurred. The entrapped enzyme fraction was subjected to the spectrophotometric analysis to determine its activity and selected for further decolorization study. Immobilization efficiency was calculated as the ratio of the enzyme entrapped (difference between the enzyme loaded and the enzyme in the supernatant after washing × 100).

### Reusability and shelf life

The reusability of the immobilized MnP was investigated in a batch experiment using MnSO_4_ as a substrate. The gel entrapped MnP was incubated with 1 mM MnSO_4_ in 50 mM sodium malonate buffer at room temperature (25°C). At the end of each cycle, sol–gel entrapped biocatalyst washed three times with the same buffer before treating the fresh substrate solution. The remaining activity was calculated relative to the initial MnP activity after each cycle. For the commercial utilization of industrial enzymes shelf life is an important consideration. To investigate the storage stabilities, free and immobilized MnP were incubated at room temperature (25°C) for up to 75 periodic days. After every 15 days the residual activities were measured using standard assay protocol as described earlier.

### Decolorization of textile effluents by immobilized MnP

To investigate the decolorization applicability of sol-gel-entrapped MnP, four different textile industry wastewater effluents were collected from Magna, Crescent, Arzoo, and Chenab textile industries of Faisalabad. The working conditions of a single continuous operation were: triplicate flasks containing 5 g of sol-gel-entrapped bio-catalyst (MnP), 100 mL of each textile effluent with 1 mL of 1 mM MnSO_4_ as MnP mediator, and incubated in a temperature-controlled shaker (120 rpm) for 4 h reaction time. At the end of each h, samples were collected from each flask to determine the percentage enzymatic color removal of textile effluent by considering the initial and final absorbance of treated and untreated effluents. All the collected samples were centrifuged at 5,000 g for 15 min at room temperature (25°C) and clear supernatants were analyzed spectrophotometrically. Decolorization of effluents was determined by a reduction in optical density at the wavelength of maximum absorbance of each effluent by UV-Vis spectrophotometer.

### Toxicity analysis

#### Formaldehyde and nitro amines

A spectrophotometric method was used to determine the formaldehyde in treated effluents. The method was based on formaldehyde reaction with chromo-tropic acid in the presence of magnesium sulfate producing a stable complex Mg^2+^/ cyclotetrachromotropylene. Beer’s Law is obeyed in a concentration range of 3 to 11 mg/L of formaldehyde with a correlation coefficient of 0.999. The color complex formed was analyzed by UV/Vis spectrophotometer at 535 nm (λmax). Treated and untreated wastewater sample was examined through high performance liquid chromatography (HPLC) method, using meta-nitro phenol as internal standard (IS) with a variable-wavelength UV detector.

### Statistical analysis

All the experimental data was statistically analyzed using the statistical software Minitab, version 15. The means and standard errors of means (mean ± S.E.) were computed for each treatment and S.E. values have been displayed as Y-error bars in figures.

## Competing interests

The authors declare that they have no competing interests.

## Authors’ contributions

HMNI (Research Associate of the project) participated in carrying out the experimental work on microbial cultivation, MnP production, extraction, purification, Sol-gel immobilization and its application for the decolorization of various textile industry effluents. All the research work was carried out under the supervision of MA (Principal Investigator of the project), who designed the project and supervised all the experimental and analytical work. All authors read and approved the final version of the manuscript.

## References

[B1] Stoilova I Krastanov A Stanchev V Properties of crude laccase from *Trametes versicolor* produced by solid-substrate fermentation Adv Biosci Biotechnol 2010 1 208 215 10.4236/abb.2010.13029

[B2] Asgher M Iqbal HMN Enhanced catalytic features of sol–gel immobilized MnP isolated from solid state culture of *Pleurotus ostreatus* IBL-02 Chin Chem Lett 2013 24 344 346 10.1016/j.cclet.2013.02.019

[B3] Asgher M Iqbal HMN Characterization of a novel manganese peroxidase purified from solid state culture of *Trametes versicolor* IBL-04 BioRes 2011 6 4317 4330

[B4] Asgher M Iqbal HMN Irshad M Characterization of purified and xerogel immobilized novel lignin peroxidase produced from *Trametes versicolor* IBL-04 using solid state medium of corncobs BMC Biotechnol 2012 12 46 10.1186/1472-6750-12-46 22862820 PMC3442999

[B5] Asgher M Kamal S Iqbal HMN Improvement of catalytic efficiency, thermo-stability and dye decolorization capability of *Pleurotus ostreatus* IBL-02 laccase by hydrophobic Sol-gel entrapment Chem Cent J 2012 6 1 110 10.1186/1752-153X-6-110 23021344 PMC3541985

[B6] Asgher M Ahmad Z Iqbal HMN Alkali and enzymatic delignification of sugarcane bagasse to expose cellulose polymers for saccharification and bio-ethanol production Ind Crops Prod 2013 44 488 495

[B7] Papinutti V Forchiassin F Lignocellulolytic enzymes from *Fomes sclerodermeus* growing in solid-state fermentation J Food Eng 2007 81 1 54 59 10.1016/j.jfoodeng.2006.10.006

[B8] Asgher M Jamil F Iqbal HMN Bioremediation potential of mixed white rot culture of *Pleurotus ostreatus* IBL-02 and *Coriolus versicolor* IBL-04 for textile industry wastewater J Bioremed Biodegrad 2012 S1 007 10.4172/2155-6199.S1-007

[B9] Takano M Nakamura M Yamaguchi M Glyoxal oxidase supplies hydrogen peroxide at hyphal tips and on hyphal wall to manganese peroxidase of white-rot fungus *Phanerochaete crassa* WD1694 J Wood Sci 2010 56 4 307 313 10.1007/s10086-009-1105-6

[B10] Saratale RG Saratale GD Chang JS Govindwar SP Outlook of bacterial decolorization and degradation of azo dyes: A review J Taiwan Inst Chem Eng 2011 42 138 157 10.1016/j.jtice.2010.06.006

[B11] Ahmed I Zia MA Iftikhar T Iqbal HMN Characterization and detergent compatibility of purified protease produced from *Aspergillus niger* by utilizing agro wastes BioRes 2011 6 4 4505 4522

[B12] Xiao H Huang J Liu C Jiang D Immobilization of laccase on amine-terminated magnetic nano-composite by glutaraldehyde crosslinking method Trans Nonferrous Met Soc China 2006 16 s414 s418

[B13] Kunamneni A Camarero S García-Burgos C Plou FJ Ballesteros A Alcalde M Engineering and applications of fungal laccases for organic synthesis Microb Cell Fact 2008 7 32 10.1186/1475-2859-7-32 19019256 PMC2613868

[B14] Yinghui D Qiuling W Shiyu F Laccase stabilization by covalent binding immobilization on activated polyvinyl alcohol carrier Lett Appl Microbiol 2002 35 6 451 456 10.1046/j.1472-765X.2002.01196.x 12460423

[B15] Maas R Chaudhari S Adsorption and biological decolorization of azo dye reactive red 2 in semicontinuous anaerobic reactors Proc Biochem 2005 40 699 705 10.1016/j.procbio.2004.01.038

[B16] Verma P Madamwar D Production of ligninolytic enzymes for dye decolorization by cocultivation of white-rot fungi *Pleurotus ostreatus* and *Phanerochaete chrysosporium* under solid-state fermentation App Biochem Biotechnol 2002 102 1 109 118 10.1385/abab:102-103:1-6:109 12396115

[B17] Wariishi H Valli K Gold MH Manganese (II) oxidation by manganese peroxidase from the basidiomycete *Phanerochaete chrysosporium*. Kinetic mechanism and role of chelators J Biol Chem 1992 267 23688 23695 1429709

[B18] Bradford MM A rapid and sensitive method for quantification of microgram quantities of protein utilizing the principle of protein dye binding Anal Biochem 1976 72 248 254 10.1016/0003-2697(76)90527-3 942051

[B19] Laemmli UK Cleavage of structural proteins during assembly of head of bacteriophage T4 Nature 1970 227 680 685 10.1038/227680a0 5432063

